# Performance-based assessment in the 21st century: when the examiner is a machine

**DOI:** 10.1007/s40037-020-00647-4

**Published:** 2021-01-11

**Authors:** Brian D. Hodges

**Affiliations:** Toronto General Hospital, University Health Network, Toronto, Canada

In their clever paper ‘Scenes, symbols and social roles: raising the curtain on OSCE performances,’ Gormley and colleagues [1] raise some challenging questions about OSCEs. Using a lens of symbolic interactionism [2] and the perspective of Canadian sociologist Erving Goffman, they argue that “the self is multifaceted, and capable of performing and producing different aspects of oneself depending on the situation or encounter.” They tell us then, that we need to worry about the difference between students “performing” (for credit) and “being” (presumably a more authentic version of themselves) [1]. I agree with Gormley and team, but my concerns go even deeper. I see a coming paradigm shift in performance-based assessments away from human-rated scenarios to examinations in which human judgment is first distanced and made anonymous and then augmented or replaced by artificial intelligence (AI).

I agree with Gormley and colleagues that if students are forced to choose what aspects of the self to portray for the examiners watching them, then there are urgent questions to be asked regarding the work they do on themselves to try to conform to an imagined ideal. Indeed, there has been evidence for some time that OSCEs, multiple mini interviews (MMIs) and other forms of performance-based assessment can drive students toward maladaptive or stereotypical performances.

These performance distortions run the gamut from the so-called ‘shotgun’ interview that arises from the overuse of checklists by evaluators [3] to the production of pseudo-empathy when scenarios call for a display of emotion that is not genuinely felt [4]. Perhaps more profoundly, anthropologist Janelle Taylor questions the ethical work done in simulation, citing as an example a student interviewing an actor who cannot in real life afford the very treatment that is being discussed with the ‘patient’ she is portraying [5]. And concerningly, in work led by Saleem Razack, we found that students taking an MMI for medical school admissions learned new accents, practiced emotional responses to disguise their ethnicity and worked to decode the ways of being that they believed would impress on examiners that they were the ‘right kind of person’ for medical school [6].

## Surveillance and the power of a panopticon

Fundamentally all forms of examination are surveillance, but OSCEs and MMIs are what philosopher Michel Foucault called a *panopticon*: a surveillance technology that has the power to shape an individual’s behaviour toward a norm through constant, often anonymous, observation [7]. Unlike examinations of knowledge however, in all performance-based examinations, physical appearance matters. While some institutions prescribe a standardized outfit, such as dark slacks and a white shirt for both men and women, clothing is only a superficial aspect of the myriad personal qualities, gestures and markers of identity. So, it is perhaps not surprising, albeit concerning, that students try to amplify or suppress particular outward manifestations of gender, sexuality, culture, religion, language or disability/ability when trying to perform what they perceive to be the desired version of themselves.

Until recently, this work on the self has been designed to respond to a human examiner (and standardized patient) physically present with the examinee. In such situations, a student tries to *persuade* through their performance. However, as video cameras are increasingly deployed and the data streaming from them is analyzed using AI-assisted pattern recognition technologies, the social dynamics of examination will be completely altered.

## When the examiner is a machine

With the dawn of AI, the examining gaze will no longer be exclusively human. Already high schools in China have equipped classrooms with cameras that can recognize the emotions of students, take attendance and track what students are doing in class [8]. Computer systems can detect “subtle ‘micro-expressions’ and behavioral characteristics associated with joy, trust, fear, surprise, sadness, disgust, and anger” that, according to marketing materials, eliminate “human bias” according to one California-based company that goes on to recommend such systems for law enforcement and education alike [9].

It is already common practice to use cameras in national medical licensure examinations, and it cannot be long before images streaming from clinics and wards are also used for workplace-based assessment. For the moment, most such examinations are human rated, but increasingly AI and machine learning will augment pattern recognition and perhaps begin to replace human judgment.

Hanson predicted this shift in technology and the implications of shifting from examinations that are interactional to those that embed anonymized surveillance. He wrote,

*If the artful presentation of Goffman’s self is seductive, what happens in testing *[that involves anonymous observation] *is, to borrow a simile from Jean Baudrillard, pornographic. Pornography differs from seduction in that the individual fixed by the pornographic gaze is powerless to conceal, control, or nuance anything. She or he is displayed for the observer’s inspection …* [10]

In writing this I want to underscore that I see many positive benefits to the rise of simulation and performance-based assessment in the last 50 years. Performance-based assessments encourage students to give more attention to communication and interpersonal skills at a time when caring and compassion are strained in our harried healthcare institutions. Similarly, requiring students to demonstrate physical examination and other skills has led to progress in assuring learners have psychomotor skills to transfer into real, clinical workplaces.

Yet as technologies emerge that allow behavioural observation at a distance and the categorization of patterns of human behaviours by AI evolves, the ethical dimensions of assessment will shift substantially. Virginia Eubanks in her book *Automating Inequality* writes that we must be vigilant in designing anonymized, algorithm-driven judgments. For medical education, this will go well beyond our 20th century debates about checklists versus global ratings. I share the worry of Gormley and colleagues that old fashioned, poorly designed, human-administered checklists are in tension with good medical practice. Yet I am more preoccupied with the spectre of extra-local, anonymized observation using AI algorithms to judge behaviours. Or more simply put, the computer that scrutinizes you when you pass through airport security to ensure that your expressions, behaviours and emotional tone conform to expected norms may be coming to a medical education examination centre very soon.

The world is riddled with biased systems of judgment. As Eubanks warns “When automated decision-making tools are not built to explicitly dismantle structural inequities, their speed and scale intensify them” [11]. This is surely a challenge of the first order as we build and refine our medical education panopticons for the 21st century.
